# Prioritising respiratory syncytial virus prevention in low-income and middle-income countries

**DOI:** 10.1016/S2214-109X(23)00165-1

**Published:** 2023-05

**Authors:** Xavier Carbonell-Estrany, Eric AF Simões, Louis J Bont, Bosco A Paes

**Affiliations:** Neonatology, Hospital Clinic, Barcelona 08036, Spain; Department of Pediatrics, University of Colorado School of Medicine, Aurora, CO, USA; Department of Epidemiology, Center for Global Health, Colorado School of Public Health, Aurora, CO, USA; Laboratory of Translational Immunology and Department of Paediatrics, Wilhelmina Children’s Hospital, University Medical Centre Utrecht, Utrecht, Netherlands; Department of Pediatrics, McMaster University, Hamilton, ON, Canada

Respiratory syncytial virus is a major cause of lower respiratory tract infection morbidity and mortality in children globally, causing 3·2–3·6 million hospitalisations and more than 100 000 deaths annually in children younger than 5 years, 99% of which occur in low-income and middle-income countries (LMICs).^1^ Despite being identified 65 years ago, at the same time as poliovirus, there is no widely available and affordable preventive strategy for respiratory syncytial virus.

Over the past few years, the advent of several preventive interventions^2^ finally provides opportunities to address the burden of respiratory syncytial virus disease. A long-acting, single-dose monoclonal antibody that provides season-long protection was approved in Europe in October, 2022.^2^ Additionally, the interim results of phase 2 and phase 3 maternal respiratory syncytial virus vaccine trials for the prevention of severe lower respiratory tract infection caused by respiratory syncytial virus in infants, among whom the burden is greatest, are promising, although the studies await peer review.^3^ Vaccines for use in early childhood are also under clinical investigation.^2^ However, in the past, new vaccines to prevent important respiratory and diarrhoeal illnesses were authorised and implemented first in high-income countries (HICs) and much later in LMICs. Adoption in LMICs, in our view, lags unacceptably behind. For example, the *Haemophilus influenzae* type b conjugate vaccine was implemented in LMICs almost 20 years after it was introduced in HICs.^4^ Similarly, the pneumococcal conjugate vaccine, rapidly adopted in HICs, took 15 years to achieve 59% utilisation in middle-income countries.^5^ Finally, despite the highly successful rotavirus vaccine roll-out in LMICs in 2009, as of January, 2022, it remained unavailable in 21% of African countries and 54% of southeast Asian countries, two regions that account for the majority of rotavirus mortality.^6^

The disparity in the implementation of highly effective preventive interventions in LMICs, where most mortality from respiratory and diarrhoeal illnesses occurs, is unacceptable and demands urgent concerted action of the global community. This includes the pharmaceutical industry, the UN, public health agencies, foundations, governments and non-governmental organisations, regional and national immunisation technical advisory groups, economists, paediatricians, allied health-care and research personnel, and, importantly, family groups, who strongly advocate for our most vulnerable infants that are least able to advocate for themselves.

Several barriers preclude the successful delivery of respiratory syncytial virus preventives in LMICs, including low awareness; a lack of country-specific burden data; logistical, administrative, and structural constraints; and the cost of the products.^7,8^ Clearly, the first step must be equitable access to new interventions. Accessibility to respiratory syncytial virus preventives must be streamlined through collaboration, such as that established between the Bill & Melinda Gates Foundation and Pfizer, which will enable faster and more equitable deployment of the maternal respiratory syncytial virus vaccine to LMICs.^9^ This collaboration is essential to dissolve inequity and accelerate progress. Second, awareness must be increased by disseminating information on the impact of respiratory syncytial virus infection on young children, its manifestations and therapeutic support, and the fundamentals of prevention. Third, it is imperative to address the shortfall in diagnostic tools and clinical resources, particularly the basic need for diagnostic tests to confirm respiratory syncytial virus infection and measure its country-specific impact, and effective oxygen delivery and oximeters. Improved diagnosis is currently supported by WHO and the Gates Foundation. Fourth, the experience, knowledge, and expertise of Gavi, the Vaccine Alliance, and UNICEF, as well as that gained during the COVID-19 pandemic, will be crucial. Fifth, intrinsic to the challenges faced in LMICs is the issue of cost (appendix). Implementing new strategies will have a cost that these countries, undoubtedly, cannot meet alone. A respiratory syncytial virus vaccine price of less than US$5 per dose has been set as a target for LMICs. Importantly, new respiratory syncytial virus preventives involve a single dose (vs three or four doses for pneumococcal conjugate vaccine). In 2011, Gavi successfully negotiated a reduced price of human papillomavirus vaccines via a public–private partnership in LMICs.^10^ The simultaneously revised and simplified dosing schedule by WHO facilitated roll-out and further reduced overhead costs. Nevertheless, the uptake of human papillomavirus vaccines remains low, highlighting the challenges faced. Finally, the introduction of any new intervention must be preceded by engagement of local communities to determine preferences, understanding, and priorities.

We have now reached a pivotal point in respiratory syncytial virus prevention that demands international recognition, collaboration, and full commitment and engagement of key stakeholders to effect change. The new respiratory syncytial virus preventive strategies have the potential to substantially improve child health and reduce morbidity and mortality, but to truly realise their full potential, the outlined barriers in LMICs must be urgently addressed. We put forward that the time to act is now. The first step should be to bring the key stakeholders together to agree on a unified plan of action to finally end the devastating burden of respiratory syncytial virus in children residing in LMICs.

## Supplementary Material

Supplementary Appendix

## Appendix

† The RSV Prevention Collaborators: Adaeze Ayuk (Department of Paediatrics, College of Medicine, University of Nigeria, Enugu, Nigeria and Department of Paediatrics, University of Nigeria Teaching Hospital, Ituku Ozalla Enugu, Nigeria); Angela Gentile (Epidemiology Department, Ricardo Gutiérrez Children’s Hospital, University of Buenos Aires, Buenos Aires, Argentina); Anne Greenough (Department of Women and Children’s Health, School of Life Course Sciences, Faculty of Life Sciences and Medicine, King’s College London, London, UK); Antonio Moreno-Galdó (Department of Paediatrics, Paediatric Allergy and Pulmonology Section, Vall d’Hebron University Hospital, Autonomous University of Barcelona, Barcelona, Spain and Center for Biomedical Research on Rare Diseases [CIBERER], Carlos III Health Institute, Madrid, Spain); Sudha Basnet (Department of Pediatrics, Institute of Medicine, Tribhuvan University, Kathmandu, Nepal); Arun Sharma (Department of Pediatrics, Institute of Medicine, Tribhuvan University, Kathmandu, Nepal); Asuncion Mejias (Department of Infectious Diseases, St Jude Children’s Research Hospital, Memphis, TN, USA); Octavio Ramilo (Department of Infectious Diseases, St Jude Children’s Research Hospital, Memphis, TN, USA); Barry Rodgers-Gray (Violicom Medical Limited, Aldermaston, UK); Bernhard Resch (Department of Pediatrics, Medical University of Graz, Graz, Austria); Brigitte Fauroux (Pediatric Noninvasive Ventilation and Sleep Unit, AP-HP, Necker–Enfants Malades Hospital, Paris, France and Research unit EA7330 VIFASOM, Paris University, Paris, France); Carlos E Rodriguez-Martinez (Department of Pediatrics, School of Medicine, National University of Colombia, Bogota, Colombia and Department of Pediatric Pulmonology, School of Medicine, El Bosque University, Bogota, Colombia); Chadi El Saleeby (Pediatric Infectious Diseases and Hospital Medicine, MassGeneral Hospital for Children, Harvard Medical School, Boston, MA, USA); W Charles Huskins (Division of Pediatric Infectious Diseases, Mayo Clinic, Rochester, MN, USA); Cheryl Cohen (Centre for Respiratory Disease and Meningitis, National Institute for Communicable Diseases, Johannesburg, South Africa and School of Public Health, University of the Witwatersrand, Johannesburg, South Africa); Harish Nair (Centre for Global Health, Usher Institute, Edinburgh Medical School, University of Edinburgh, Edinburgh, UK and School of Public Health, University of the Witwatersrand, Johannesburg, South Africa); Shabir Madhi (Vaccines and Infectious Diseases Analytics Unit and African Leadership in Vaccinology Expertise, University of the Witwatersrand, Johannesburg, South Africa); David Greenberg (The Pediatric Infectious Disease Unit, Soroka University Medical Center, Beer-Sheva, Israel and Faculty of Health Sciences, Ben-Gurion University of the Negev, Beer-Sheva, Israel); Ron Dagan (Faculty of Health Sciences, Ben-Gurion University of the Negev, Beer-Sheva, Israel); Eugenio Baraldi (Department of Women’s and Children’s Health, University Hospital of Padova, Padova, Italy and Institute of Pediatric Research “Città della Speranza”, Padova, Italy); Evan J Anderson (Departments of Medicine and Pediatrics, Emory University School of Medicine, Atlanta, GA, USA); Federico Martinon-Torres (Pediatrics Department, Santiago Clinic Hospital CHUS, Santiago de Compostela, Spain and Health Research Institute of Santiago, University of Santiago de Compostela, Santiago de Compostela, Spain); Fernando F Polack (Fundacion INFANT, Buenos Aires, Argentina); Giovanni Piedimonte (Department of Pediatrics, Biochemistry and Molecular Biology, Tulane University, New Orleans, LA, USA); Hayley A Gans (Department of Pediatrics, Stanford University Medical Center, Stanford, CA, USA and Pediatric Infectious Diseases Program for Immunocompromised Hosts, Lucile Packard Children’s Hospital, Stanford Children’s Health, Centre for Academic Medicine, Palo Alto, CA, USA); Heather J Zar (Department of Paediatrics and Child Health, Red Cross Children’s Hospital, Cape Town, South Africa and Medical Research Council Unit on Child and Adolescent Health, University of Cape Town, Cape Town, South Africa); Hiroyuki Moriuchi (Department of Pediatrics, Graduate School of Biomedical Sciences, Nagasaki University, Nagasaki, Japan and School of Tropical Medicine and Global Health, Nagasaki University Hospital, Nagasaki, Japan); Hitoshi Oshitani (Department of Virology, Tohoku University Graduate School of Medicine, Sendai, Japan); Ian Mitchell (Cumming School of Medicine, University of Calgary, Calgary, Canada and Department of Paediatrics, Alberta Children’s Hospital, Calgary, Alberta, Canada); D James Nokes (Kenya Medical Research Institute-Wellcome Trust Research Programme, Centre for Geographic Medicine Research-Coast, Kilifi, Kenya and School of Life Sciences, University of Warwick, Coventry, UK); Patrick Munywoki (Kenya Medical Research Institute-Wellcome Trust Research Programme, Centre for Geographic Medicine Research-Coast, Kilifi, Kenya); Jarju Sheikh (Molecular Diagnostic Laboratory, Medical Research Council Unit The Gambia at London School of Hygiene and Tropical Medicine, Banjul, The Gambia); Jeffrey Pernica (Department of Pediatrics, McMaster University, Hamilton, ON, Canada); Jesse Papenburg (Department of Pediatrics and Department of Epidemiology, Biostatistics and Occupational Health, McGill University, Montreal, QC, Canada); Joan Robinson (Department of Pediatrics, University of Alberta, Edmonton, AB, Canada); Joanne De Jesus-Cornejo (Medical Department, Pediatrics, Research Institute for Tropical Medicine, Muntinlupa, Philippines); Joanne Langley (Faculty of Medicine and Department of Pediatrics, Dalhousie University, Halifax, NS, Canada and Canadian Center for Vaccinology (Dalhousie University, IWK Health and Nova Scotia Health), Halifax, NS, Canada; Johannes Liese (Department of Pediatrics, University Hospital Würzburg, Würzburg, Germany); Josep Figueras-Aloy (Neonatology, Hospital Clinic, Barcelona, Spain); Quique Bassat (ISGlobal, Hospital Clinic, Barcelona, Spain and Catalan Institution for Research and Advanced Studies [ICREA], Barcelona, Spain); Juan Pablo Torres (Department of Pediatrics, Division of Pediatric Infectious Diseases, Universidad de Chile, Santiago, Chile); Kathryn Edwards (Department of Pediatrics, Vanderbilt University Medical Center, Nashville, TN, USA); Leonard Krilov (Department of Pediatrics, NYU Long Island School of Medicine, Mineola, NY, USA and Department of Pediatrics, NYU Langone Hospital-Long Island, Mineola, NY, USA); Maduja Divaratne (Department of Microbiology, Faculty of Medicine, University of Peradeniya, Peradeniya, Sri Lanka); Manuel Sánchez Luna (Neonatology Department, Hospital General Universitario Gregorio Marañón, Madrid, Spain); Marcello Lanari (Alma Mater Studiorum - University of Bologna, Pediatric Emergency Unit, Department of Medical & Surgical Sciences, IRCCS-S Orsola Hospital, Bologna, Italy); Marcelo Scotta (Hospital Moinhos de Vento, Porto Alegre, Brazil and School of Medicine, Pontifical Catholic University of Rio Grande do Sul, Porto Alegre, Brazil); Renato Stein (School of Medicine, Pontifical Catholic University of Rio Grande do Sul, Porto Alegre, Brazil); Maria Ahuoiza Garba (Department of Paediatrics, Ahmadu Bello University, Zaria, Nigeria); Masaaki Mori (Department of Lifetime Clinical Immunology, Tokyo Medical and Dental University, Tokyo, Japan and Department of Internal Medicine, St. Marianna University School of Medicine, Kanagawa, Japan); Merih Cetinkaya (Department of Pediatrics, Health Sciences University, Basaksehir Cam and Sakura City Hospital, Istanbul, Turkey); Mitchell Goldstein (Department of Pediatrics, Loma Linda University Children’s Hospital, Loma Linda, CA, USA); Najwa Khuri-Bulos (Pediatrics and Infectious Disease, University of Jordan, Amman, Jordan); Nestor E Vain (Department of Pediatrics, School of Medicine, University of Buenos Aires, Buenos Aires, Argentina and Newborn Medicine, Hospitals Sanatorio Trinidad Palermo, San Isidro and Ramos Mejía, Buenos Aires, Argentina); Nikolaos Papadopoulos (Allergy Department, University of Athens, Athens, Greece and Lydia Becker Institute, University of Manchester, Manchester, UK); Nusrat Homaira (Centre for Child Health Research and Innovation [ChERI], University of New South Wales, Randwick, NSW, Australia and Respiratory Department, Sydney Children’s Hospital, Randwick, NSW, Australia); Paolo Manzoni (School of Medicine, University of Turin, Turin, Italy and Department of Maternal-Infant Medicine, Degli Infermi Hospital, Ponderano, Italy); Pedro A Piedra (Department of Molecular Virology and Microbiology, and Pediatrics, Baylor College of Medicine, Houston, TX, USA); Peter Moschovis (Divisions of Pediatric Pulmonary Medicine and Global Health, Massachusetts General Hospital, Harvard Medical School, Boston, MA, USA); Peter Openshaw (National Heart and Lung Institute, Imperial College London, London, UK); Richard Thwaites (Neonatal Unit, Royal Stoke University Hospital, Stoke-On-Trent, UK); Rohitha Muthugala (Department of Virology, National Hospital Kandy, Kandy, Central Province, Sri Lanka); Rolando Ulloa-Gutierrez (Department of Infectious Diseases, Children National’s Hospital “Dr. Carlos Sáenz Herrera”, Caja Costarricense de Seguro Social [CCSS], San José, Costa Rica and Faculty of Medicine, University of Medical Sciences [UCIMED], San José, Costa Rica); Rosa Rodriguez Fernandez (Gregorio Marañón Health Research Institute [IiSGM], Maternal and Child Hospital Gregorio Marañón, Madrid, Spain); Satoshi Kusuda (Department of Pediatrics, Kyorin University, Tokyo, Japan); Shobha Broor (Department of Microbiology, All India Institute of Medical Sciences, New Delhi, India); Simon B Drysdale (Centre for Neonatal and Paediatric Infection, St George’s University of London, London, UK and Department of Paediatrics, St George’s University Hospitals NHS Foundation Trust, London, UK); Terho Heikkinen (Department of Pediatrics, University of Turku and Turku University Hospital Turku, Finland); Vasanthi Avadhanula (Department of Molecular Virology and Microbiology, Baylor College of Medicine, Houston, TX, USA); Xavier Saez-Llorens (Department of Infectious Diseases, Children’s Hospital Doctor José Renán Esquivel and Cevaxin Clinical Research Center, Panama City, Panama); Xin Wang (Department of Biostatistics, School of Public Health, Nanjing Medical University, Nanjing, Jiangsu, China); You Li (Department of Epidemiology, School of Public Health, Nanjing Medical University, Nanjing, Jiangsu, China); Joseph L Mathew (Department of Pediatrics, Post Graduate Institute of Medical Education & Research [PGIMER], Chandigarh, India).
