# Supplementary material for: Prioritising respiratory syncytial virus prevention in low-income and middle-income countries
Source: Lancet Glob Health. Author manuscript; Available in PMC 2024 May 8. (PMC11077509; doi:10.1016/S2214-109X(23)00165-1)

THE LANCET
Global Health

Supplementary appendix

This appendix formed part of the original submission and has been peer reviewed. We post it as supplied by the authors.

Supplement to: Carbonell-Estrany X, imões EAF, Bont LJ, Paes BA, on behalf of the RSV Prevention Collaborators. Prioritising respiratory syncytial virus prevention in low-income and middle-income countries. *Lancet Glob Health* 2023; **11:** e655–57.

Cost remains a central issue and LMICs will require support from the global community to reduce the devastating impact of RSV

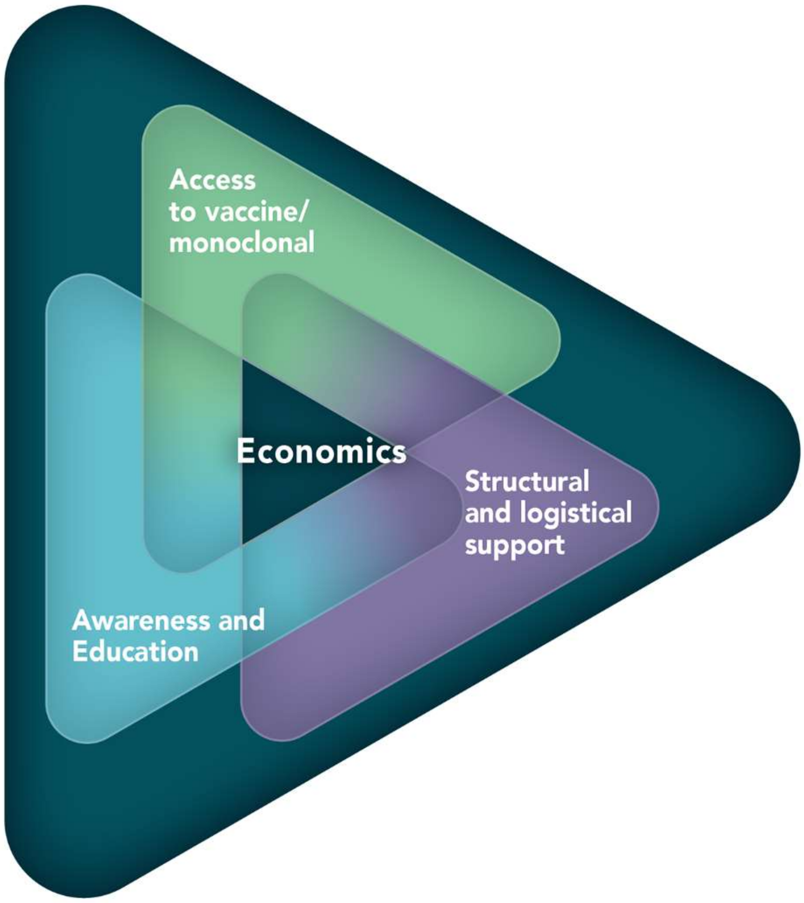

Supplement: Supplementary Appendix [file NIHMS1982417-supplement-Supplementary_Appendix.pdf]
